# A study on the immunological basis of the dissociation between type I-hypersensitivity skin reactions to *Blomia tropicalis *antigens and serum anti-*B. tropicalis *IgE antibodies

**DOI:** 10.1186/1471-2172-12-34

**Published:** 2011-06-01

**Authors:** João CM Ponte, Samuel B Junqueira, Rafael V Veiga, Mauricio L Barreto, Lain C Pontes-de-Carvalho, Neuza M Alcântara-Neves

**Affiliations:** 1 Departamento de Biointeração, Instituto de Ciências da Saúde, Universidade Federal da Bahia, Av. Reitor Miguel Calmon, Sem no. Canela, Salvador, 40110-100, Bahia, Brazil; 2 Instituto de Saúde Coletiva, Universidade Federal da Bahia, Rua Pe Feijó, 29, Canela Salvador, 40110-170, Bahia, Brazil; 3 Laboratório de Patologia e Biologia Interativa, Centro de Pesquisas Gonçalo Moniz, Fundação Oswaldo Cruz, Rua Waldemar Falcão, 121, Brotas, Salvador, 40285-885, Bahia, Brazil

## Abstract

**Background:**

Two conditions are used as markers of atopy: the presence of circulating anti-allergen IgE antibodies and the presence of positive skin prick test (SPT) reactions to allergenic extracts. The correlation between these conditions is not absolute. This study aimed at investigating immunological parameters that may mediate this lack of correlation. Individuals whose sera contained anti-*B. tropicalis *extract IgE antibodies (α*-Bt*E IgE) were divided into two groups, according to the presence or absence of skin reactivity to *B. tropicalis *extract (*Bt*E). The following parameters were investigated: total IgE levels; α*-Bt*E IgE levels; an arbitrary α*-Bt*E IgE/total IgE ratio; the proportion of carbohydrate-reactive α*-Bt*E IgE; the proportion of α*-Bt*E IgE that reacted with *Ascaris lumbricoides *extract (*Al*E); the production of IL-10 by *Bt*E- and *Al*E-stimulated peripheral blood cells (PBMC).

**Results:**

Total IgE levels were similar in the two groups, but α*-Bt*E IgE was significantly higher in the SPT-positive group (SPT**+**). A large overlap of α*-Bt*E IgE levels was found in individuals of both groups, indicating that these levels alone cannot account for the differences in SPT outcome. Individuals of the two groups did not differ, statistically, in the proportion of α-*Bt*E IgE that reacted with carbohydrate and in the production of IL-10 by *Bt*E- and *Al*E-stimulated PBMC. Both groups had part of α-*Bt*E IgE activity absorbed out by *Al*E, indicating the existence of cross-reactive IgE antibodies. However, the α-*Bt*E IgE from the SPT-negative individuals (SPT-) was more absorbed with *AlE *than the α-*Bt*E IgE from the SPT+ individuals. This finding may be ascribed to avidity differences of the α-*Bt*E IgE that is present in the two groups of individuals, and could occur if at least part of the α-*Bt*E IgE from the SPT- individuals were elicited by *A. lumbricoides *infection.

**Conclusion:**

The present results suggest that a low ratio of specific IgE to total IgE levels (in a minority of individuals), and differences in α-*Bt*E IgE avidities (which would have high affinities for *A. lumbricoides *antigens in SPT- than in SPT**+ **individuals) may play a role in the down-modulation of type-I hypersensitivity reaction against aeroallergens described in helminth-infected individuals.

## Background

Asthma is a complex disease whose phenotypic heterogeneity has been shown to depend both on genetic factors, such as those that underlie the appearance of atopy, and on environmental factors [1,2]. Among the environmental factors, one that is *sine qua non *in the determination of the development, severity and chronicity of allergic asthma is the contact with environmental allergens, especially those derived from indoor mites, cockroaches, pet epithelia and pollens [3,4]. Indeed, atopy is usually defined as an intrinsic condition characterized by the presence of circulating IgE antibodies against those environmental allergens (i.e., antibodies against innocuous antigens) and/or skin reactivity to the same allergens. Although a good association between the presence in the circulation of IgE antibodies and a skin prick test (SPT) positivity for the same aeroallergen has been reported, especially in high-income countries and in affluent sub-populations of developing countries [5,6], this is not always the case. Thus, a poor association between these two atopy markers in people from rural communities or non-affluent countries has been observed [7,8]. Recently, in partial disagreement with previous studies, the ISAAC research group reported dissociation between these atopy markers in both affluent and non-affluent populations [9].

Helminth infections usually accompany low economic development in the tropics, and they may play a role in establishing the discrepancy between the presence of positive SPT and allergen-specific IgE antibodies (sIgE) found in low-income communities [10]. Several hypotheses have been proposed to explain why, in some individuals with sIgE, contact of skin mast cells with allergen does not lead to their degranulation. Some of these hypotheses involve: (i) the competition of polyclonal IgE raised by helminths with sIgE for mast-cell Fcε receptors [11]; (ii) anti-allergen IgG4 antibodies blocking IgE-mediated immunity and allergic processes [12]; (iii) presence of cross-reactive IgE antibodies that react with glycoprotein carbohydrate moieties [α(1,3)-fucose and ß(1,2)-xylose on N-glycans], which are widely present in plants and invertebrates and produce clinically irrelevant antibodies [13]; (iv) the induction of regulatory CD4^+ ^CD25^+ ^Foxp3^+ ^T cells that are capable of down-regulating the allergic process [14].

In the present study, six different parameters were studied in individuals who had circulating IgE antibodies against *B. tropicalis *extract (*Bt*E) and had either a negative (SPT-negative individuals) or positive (SPT-positive individuals) result in a SPT in which *Bt*E was used as antigen (*Bt*E SPT). The studied parameters were: (i) the levels of circulating anti-*B. tropicalis *IgE antibodies (α*Bt*E IgE); (ii) the levels of total circulating IgE; (iii) an arbitrary α*Bt*E IgE/total IgE ratio; (iv) the proportion of the α*Bt*E IgE that reacted with carbohydrate antigenic determinants; (v) the production of IL-10 by *Bt*E- and *A. lumbricoides *extract (*Al*E)-stimulated blood mononuclear cells; (vi) the proportion of α*Bt*E IgE that cross-reacted with *Al*E.

IgE antibodies are usually biologically active in remarkably low concentrations [15]. The identification of the underlying factors that in some cases may lead these antibodies to fail to trigger, in the presence of antigen, the most potent of the IgE-associated effector mechanisms, namely the degranulation of mast cells, may be relevant for the development of possible therapies for allergic diseases.

## Results

### Anti-*Bt*E IgE levels and their correlation with wheal sizes of SPT reactions

**Figure 1:**
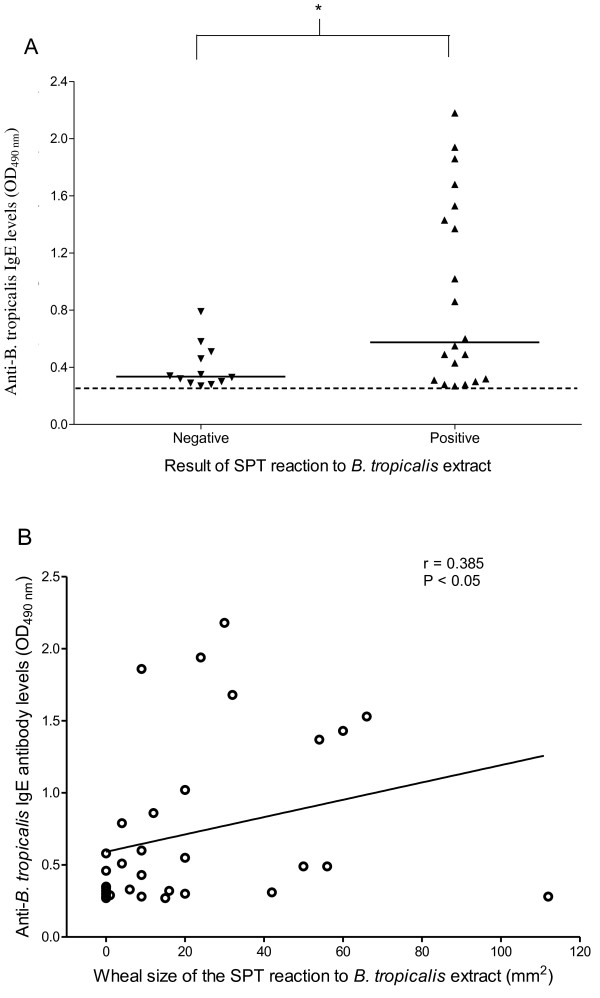
**Anti-*B. tropicalis *IgE antibody levels (A) and correlation between the sizes of the wheals produced by skin prick test (SPT) reaction to *B. tropicalis *extract and anti-*B. tropicalis *IgE antibody levels (B) in 32 individuals**. Serum anti-*B. tropicalis *IgE antibody levels and total IgE were assessed by indirect ELISA as described in the Methods section. Each symbol represents the result obtained from an individual serum. The horizontal solid lines represent the median value of the group results, and the horizontal dashed line represents the assay cut-off. *, p < 0.05, Mann-Whitney U test and Spearman's correlation.

The SPT-positive individuals had higher α*Bt*E IgE levels than the SPT-negative individuals, although a great overlap in these antibody levels could be seen between the two groups (p < 0.05; Mann-Whitney U test, Figure 1A). A weak, but statistically significant correlation (r = 0.385, Pearson's correlation; p < 0.05) was also found between α*-Bt*E IgE levels and the *Bt*E SPT wheal sizes (Figure 1 B).

### Total IgE and αBtE IgE/total IgE ratios

**Figure 2:**
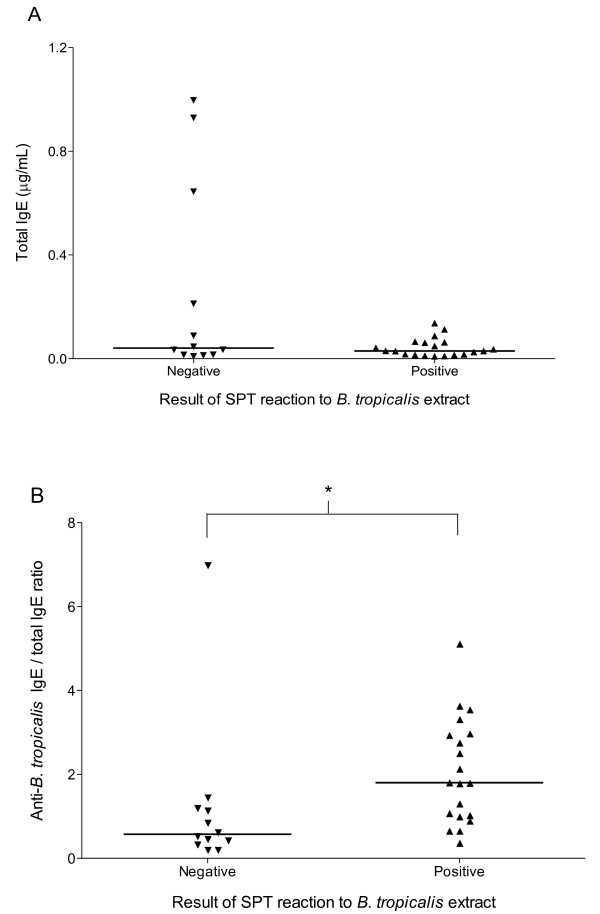
**Total serum IgE concentrations (A) and anti-*B. tropicalis *IgE/total IgE ratio (B**) **in individuals with and without positive skin prick test (SPT) results**. Serum anti-*B. tropicalis *IgE antibody and total IgE levels were assessed by indirect and capture ELISA, respectively, as described in the Methods section. Each symbol represents the result obtained from an individual serum. The horizontal solid lines represent the median value of the group results. *, p < 0.05, Mann-Whitney U test.

No statistical difference in total IgE levels was found between the groups, although a few individuals in the SPT negative group had markedly high IgE levels (Figure 2A). The α-*Bt*E IgE/total IgE arbitrary ratio was significantly smaller in the SPT negative group (p < 0.05; Mann-Whitney's U test), although the ranges of values between the two groups almost completely overlapped (Figure 2B).

### Proportion of α*Bt*E IgE that reacted with carbohydrate antigenic determinants and IL-10 production by peripheral blood cells

**Figure 3:**
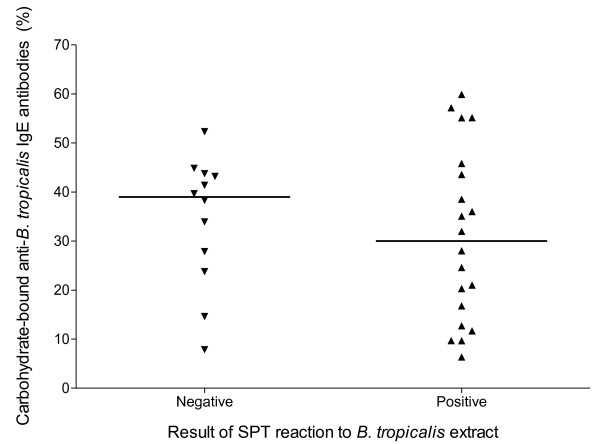
**Proportion of anti-*B. tropicalis *IgE antibodies binding to carbohydrate epitopes in individuals with and without positive skin prick test (SPT) results**. The values represent the reduction in the binding to the *B. tropicalis *extract, in an ELISA, of the antibodies, caused by pre-treating the antigen by metaperiodate/borohydride, as explained in the Methods section. Each symbol represents the result obtained from an individual serum. The horizontal lines represent the median value of the group results. There was no statistically significant difference between the two studied groups (p > 0.05, Mann-Whitney U test).

Overall, pre-treatment of the *Bt*E-solid phase with metaperiodate/borohydride led to varied degrees of reduction in the binding of IgE, allowing the determination that 10 to 60% of the α*Bt*E IgE present in the sera reacted with a metaperiodate/borohydride-sensitive antigen (Figure 3). However, no statistically significant difference was found in the proportion of α*Bt*E IgE that reacted with metaperiodate/borohydride-treated *Bt*E between the two studied groups (p > 0.05, Mann-Whitney U test).

**Figure 4:**
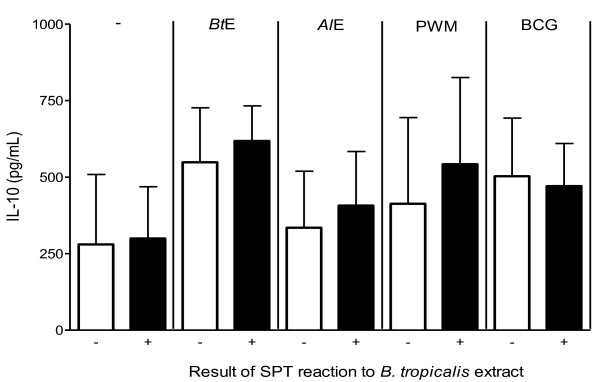
**IL-10 production by peripheral blood cells from individuals with positive or negative skin prick test (SPT) reactions to *B. tropicalis *extract (*Bt*E), as indicated in the x-axis**. Whole blood cells were assessed for their capacity of producing IL-10 *in vitro *either when non-stimulated (-) or when stimulated with *Bt*E, *A. lumbricoides *extract (*Al*E), pokeweed mitogen (PWM) or bacillus Calmette-Guérin lysate (BCG), as indicated in the top of the figure. Columns represent the mean IL-10 concentrations in the supernatants of cultures from 12 individuals with negative SPT reaction to *Bt*E or from 19 individuals with positive SPT reaction to *Bt*E. Vertical bars represent the standard deviations of the means. No statistically significant differences were found between the negative SPT and the positive SPT groups for any of the tested stimulants (p > 0.05, Mann-Whitney U test).

Blood cells from subjects of the two different groups were assessed for their capacity to produce IL-10 when cultivated without stimulus or stimulated with *Bt*E, *Al*E, bacillus Calmette-Guérin (BCG) lysate, or pokeweed mitogen (PWM) *in vitro*. No statistically significant differences between the negative SPT and the positive SPT groups were found (Figure 4).

### Cross-reactivity of IgE antibodies with *B. tropicalis *and *A. lumbricoides *antigens

**Figure 5:**
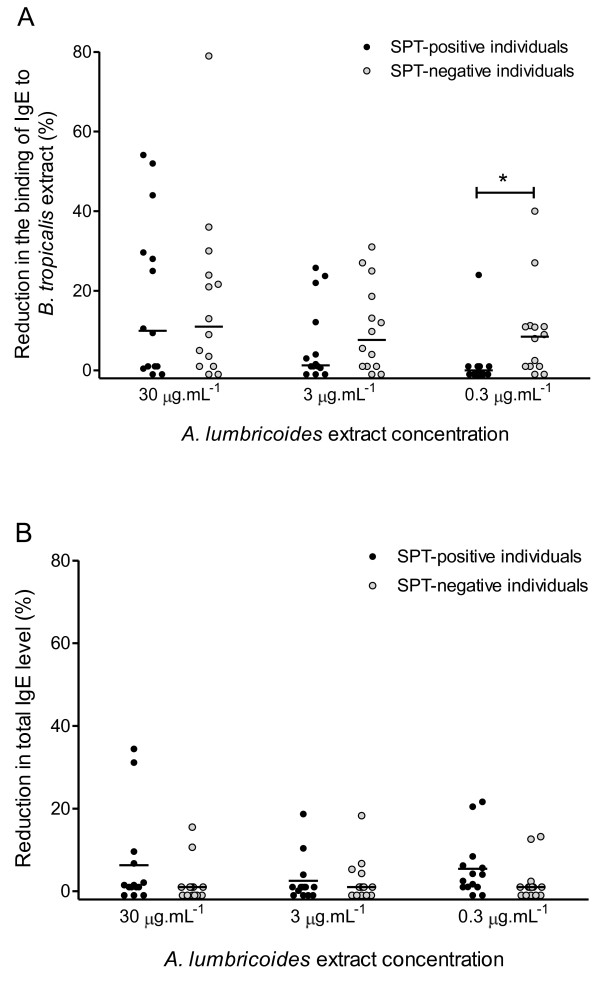
**Reaction of anti-*B. tropicalis *IgE antibodies and total IgE to *A. lumbricoides *extracts**. Sera from individuals with positive (SPT-positive individuals) or negative (SPT-negative individuals) skin prick test reactions to *B. tropicalis *extract were pre-incubated with the indicated concentrations of *A. lumbricoides *extract and tested in an indirect ELISA using *B. tropicalis *extract as antigen (**A**) or assayed for total IgE level (**B**). The reductions in anti-*B. tropicalis *IgE antibody levels and total IgE levels were calculated as described in Methods section. *, p < 0.05, Mann-Whitney U test.

Sera from SPT-positive and SPT-negative individuals were pre-incubated with increasing amounts of *Al*E before assayed for α-*Bt*E IgE or total IgE levels. Overall, more reduction in α-*Bt*E IgE levels was found in the negative SPT group than in the positive SPT group in all *Al*E concentrations. (Figure 5A). A plateau of reduction was reached when the sera were incubated with 30 μg/mL of *Al*E: larger concentrations of this extract did not lead to lower binding of the α-*Bt*E IgE to the solid-phase *Bt*E (data not shown). However, only with the lowest concentration of *Al*E (0.3 μg/mL) that was used, the difference between the groups was statistically significant (p < 0.05, Mann-Whitney U test). In most of the studied sera, the proportion of total IgE that was reduced by incubation with *Al*E (Figure 5B) was lower than the proportion of α*-Bt*E IgE that was reduced by the same treatment (Figure 5A). In some sera, in fact, the reduction in α-*Bt*E IgE level was not accompanied by any measurable reduction in total IgE level. For instance, 18 out of 28 sera (64%) had a proportion of their α*-Bt*E IgE inhibited by incubation with 30 μg/mL of *Al*E (in these 18 sera, the proportion of inhibited antibody activity ranged from 3 to 79%), whereas only 7 of these 28 sera (25%) had their total IgE levels reduced by the same treatment (in these 7 sera, the proportion of reduction in total IgE levels ranged from 2 to 34%).

## Discussion

Some aspects of the immune response that may underlie the absence of a positive *Bt*E SPT in the presence of circulating α-*Bt*E IgE were assessed in a group of poor individuals from a Brazilian northeastern large urban center. Both individuals with positive SPT and individuals with negative SPT were selected from the same area, and were probably subjected to the same social and environmental conditions, including exposition to the same pathogens. All studied subjects had serum α-*Bt*E IgE levels clearly above the cut-off of the employed assay.

The total IgE levels did not differ in the two studied groups, although the levels in three out of the 12 SPT-negative individuals were at least 4.7-fold higher than the highest total IgE value in the group of SPT-positive individuals. As there was a great overlap of total IgE levels between the two groups, a possible blocking of IgE receptors in mast cells by allergen-unrelated IgE could not explain the negativity in the SPT in the majority of the individuals. However, since distinct factors may mediate the SPT negativity in different patients, one cannot exclude that the high levels of total IgE in a minority of individuals could be inhibiting, by competition with low levels of specific IgE, the degranulation of mast cells. Indeed, when an arbitrary ratio of specific to total IgE was calculated for each serum, the ratios in two out of 12 sera from the SPT-negative individuals were smaller than the smallest ratio observed in the sera from 20 SPT-positive individuals. In these two individuals, therefore, it is possible that the negativity in the SPT could be due to the blocking of allergen-specific by non-specific IgE. A prediction of this hypothesis is that the sera from these two individuals would fail to sensitize basophils for *in vitro *allergen-triggered degranulation. A larger sample of SPT-positive and SPT-negative individuals than the one used in the present work, however, should be studied in order to allow one to conclude that a significant proportion of SPT-negative individuals have smaller specific to total IgE ratios than the SPT-positive individuals.

In the studied population, α-*Bt*E IgE levels were significantly higher in those subjects presenting with positive SPT than with negative SPT, indicating that high serum specific IgE antibody levels are associated with SPT positivity. In agreement with this finding, a statistically significant correlation, although weak, between circulating α-*Bt*E IgE levels and the mean wheal size of the SPT reactions was observed. However, as a great overlap of α-*Bt*E IgE levels was found between the SPT-negative and the SPT-positive groups (11 out of 12 sera from the negative SPT group, and 11 out of 20 sera from the positive SPT group, produced ELISA optical densities between the cut-off and 0.3), the simple explanation that low levels of specific IgE led to negative SPT in all the studied individuals does not hold up.

Allergenic cross-reactive carbohydrate determinants (CCDs) are sugar moieties of glycoproteins that induce IgE production and cross-react with a broad range of allergens [13]. CCDs have been implicated in the discrepancy between the presence of specific IgE to pollen and the absence of a positive SPT to that same allergen, as the cross-reactive IgE antibodies seem to be incapable of inducing mast cell degranulation [16]. It is therefore believed that the carbohydrate-reactive IgE antibodies are clinically irrelevant, possibly because they are unable to trigger degranulation by cross-linking the mast-cell receptors or because they have low affinity for the antigen [17]. In the present work, the pre-treatment of *Bt*E with metaperiodate/borohydride caused varying reduction levels (from 10 to 60%) in the optical densities that were obtained in the ELISA performed with sera from both SPT-positive and SPT-negative individuals. However, the difference in percentages of reduction between the positive-SPT and negative-SPT groups was not statistically significant. These carbohydrates-binding IgE antibodies, therefore, do not appear to affect the mast cell-dependent skin response to *B. tropicalis *antigens, unlike the antibodies against pollen antigens [16]. However, the possibility that the IgE antibodies from the two groups of individuals studied in the present work recognize different carbohydrate moieties in the *Bt*E, and the anti-carbohydrate IgE in the SPT-negative individuals would not cross-link mast-cell receptors in the presence of *B. tropicalis *antigens, cannot be excluded. It would, therefore, be worthwhile to investigate whether carbohydrate-binding antibodies from the two groups are equally able to sensitize basophils for antigen-triggered degranulation *in vitro*.

Helminthic infections have the ability to modulate the human immune system and suppress allergic responses, although this does not happen in infections with all helminth species [18]. With regard to asthma and atopy, the role of *A. lumbricoides *infections is controversial. In studies with Chinese and Costa Rican children, an increased risk of childhood asthma and atopy has been associated with *A. lumbricoides *infection [19,20]. However, studies with Cuban and Ethiopian children have shown that infection with this worm protects against atopic dermatitis and wheezing [21,22]. In the present work, an *A. lumbricoides *extract was used in order to study the influence of *Ascaris *antigens in two aspects of the immune response: IL-10 production and presence of cross-reactive antibodies.

It has been proposed that a balance between different populations of cytokine-producing lymphocytes, such as Th2 and Th1, and the presence of IL-10 - producing Tr1, greatly affect the response to harmless environmental molecules and the development of an allergic inflammatory response [23]. Moreover, the *in vitro *production of histamine by human mast cells, derived from umbilical cord blood, has been shown to be inhibited by IL-10 [24]. In an attempt to assess if there were differences in IL-10 production by peripheral blood cells from individuals with serum α-*Bt*E IgE, with or without positive *Bt*E SPT, their blood were cultivated in the presence of PWM, BCG lysate, *Bt*E, and *Al*E. The IL-10 production by the blood cells, subjected to any of the four stimuli, of subjects with and without positive *Bt*E SPT, did not differ. This result is consistent with the finding that mesenteric lymph node cells of IL-10^-/- ^mice that had been chronically infected with the helminth *Heligmosomoides polygyrus*, when transferred into uninfected allergen-sensitized wild-type recipients, suppressed the allergic inflammation, leading to the conclusion that the cells activated by *H. polygyrus *infection do not depend on intrinsic IL-10 to suppress the allergic response [14]. This is a controversial issue and probably depends on the species of helminth and/or of the host. For instance, Gabonese children with urinary schistosomiasis have been shown to have a lower prevalence of a positive skin reaction to house-dust mite than those without the disease, a finding that was associated to IL-10 production [25]. However, in our study population, levels of IL-10 do not appear to influence the dissociation between the presence of α-*Bt*E IgE and the absence of *Bt*E SPT. Whether further investigations, studying populations large enough to allow the groups of SPT-positive and SPT-negative individuals to be further stratified in sub-groups of individuals with high and low total IgE levels, would show differences in IL-10 production in response to either helminthic or mite antigens, is open to speculation.

Cross-reactions between helminthic and mite antigens have been reported. A study published in 2001 shows cross-reactivity between the nematode *Anisakis simplex *and the dust mites *Acarus siro, Lepidoglyphus destructor, Tyrophagus putrescentiae *and *Dermatophagoides pteronyssinus *[26]. There is little information, however, about cross-reactivities between *B. tropicalis *and *A. lumbricoides *antigens. Only one study describes cross-reactive antigens between these two organisms, namely tropomyosin and glutathione-S-transferase [27]. In accordance with these findings, cross-reactivity between *B. tropicalis *and *A. lumbricoides *antigens was demonstrated in the present study. The binding of α-*Bt*E IgE to *Bt*E was reduced in a dose-dependent manner by pre-incubation of the sera with an *A. lumbricoides *extract, in sera from both positive-SPT and negative-SPT individuals. At the highest concentrations of *Al*E (30 and 300 μg/mL), the proportion of α-*Bt*E IgE antibodies cross-reacting with *Al*E reached a plateau (data not shown), with no difference between the two studied groups. However, in the lowest concentration of *Al*E (0.3 μg/mL), more reduction in α-*Bt*E IgE levels was seen in the SPT- negative group than in the SPT-positive group. This finding cannot be ascribed to differences in pre-absorption levels of α-*Bt*E IgE, because the sera in both groups were paired for this variable. On the other hand, it could be due to a higher avidity for *A. lumbricoides *antigens of the α-*Bt*E IgE from the SPT-negative group than the α-*Bt*E IgE from the SPT-positive group. This could happen if the cross-reactive antibodies in the SPT-negative individuals were originally produced in response to an *A. lumbricoides *infection, and therefore could have a lower avidity for *B. tropicalis *antigens than antibodies elicited by the mite antigens. In any case, it is possible that these cross-reactive antibodies may contribute to the SPT negativity in some individuals of the *Bt*E SPT-negative group. The specificity of the reduction in α-*Bt*E IgE levels by incubation with *Al*E was proved by assaying the same *Al*E-treated sera for total IgE content. If IgE bound non-specifically to *Al*E, similar proportions of reduction in total IgE concentration and in α-*Bt*E IgE activity should be observed in the *Al*E-treated sera, a fact that did not occur.

## Conclusions

Overall, α-*Bt*E IgE levels were found to be directly related to positivity in the *Bt*E SPT and with the average diameter of the *Bt*E SPT wheal. Thus, the more α-*Bt*E IgE an individual has, the more likely he/she will have a positive SPT and react more intensely when coming into contact with that allergen. However, in all or in most of the studied individuals, α-*Bt*E IgE levels, total IgE levels, IL-10 production by peripheral blood cells and carbohydrate-binding IgE did not seem to play an important role in the discrepancy between the presence of specific IgE in the bloodstream and the absence of a positive SPT in the studied individuals. On the other hand, the present results are consistent with the possibilities that a low ratio of specific IgE to total IgE levels (in a minority of individuals), and difference in antigenic specificities of the α-*Bt*E IgE (which would have a high aviditity for *A. lumbricoides *antigens in *Bt*E SPT-negative individuals), may account for the discrepancy between specific IgE levels and SPT results. The results described in the present paper indicate, therefore, that different factors may be responsible or contribute to the dissociation of positive *Bt*E SPT results and the presence of α-*Bt*E IgE in the circulation. In addition, the fact that a large proportion of the IgE antibodies that reacts with *B. tropicalis *also reacts with *A. lumbricoides *antigens in a poor Latin American population may have relevance in the immunodiagnosis of atopy and even in its pathogenesis, a possibility that is only beginning to be investigated. Further studies should be implemented with a larger number of samples to investigate the possibility that the mast-cell degranulation may be inhibited by IgE cross-reacting with mite and helminthes, in order to understand the role played by these antibodies in the pathogenesis of the type-I hypersensitivity to mite antigens.

## Methods

### Serum samples, skin prick test and studied groups

Sera containing α*Bt*E IgE were obtained from blood samples collected from 32 randomly chosen individuals out of 80 individuals from a previous work, all positive for α*Bt*E IgE, who lived in a poor area of Salvador, a major city in Northeastern Brazil. These individuals answered an ISAAC phase-I questionnaire, adapted to the Portuguese language, and had their skin response to seven regional allergens, including *B. tropicalis*, assessed. SPTs were performed by the introduction of the allergenic extracts (ALK-Abelló, São Paulo, Brazil) in the skin of the right forearm of children with a disposable lancet (ALK-lancet^®^; ALK-Abelló, São Paulo, Brazil). In negative- and positive-control reactions, saline and histamine at 10 mg/mL, respectively, substituted for the allergenic extracts. Readings were done 15 minutes after the puncture. The result of the test was considered positive if the mean diameter of the wheal was ≥ 3 mm after subtraction of the mean diameter of the negative-control wheal. A negative reaction to histamine was an exclusion criterion. These 32 individuals were further divided in two groups: a group of 12 individuals with negative *Bt*E SPT results (when tested with *Bt*E, one of them had a wheal with mean diameter of 2.5 mm, two had a wheal of 2.0 mm, one had a wheal of 1.0 mm and in the other eight no wheal was formed), with ages ranging from 6 to 48 years; and a group of 20 individuals with positive *Bt*E SPT results, with ages ranging from 5 to 46 years. Other 28 sera, that were used for investigating cross-reactivity of *B. tropicalis *and *A. lumbricoides *antigens with serum IgE antibodies, were selected from a serum bank of the Laboratório de Alergia e Acarologia of the Instituto de Ciências da Saúde, Federal University of Bahia, Brazil, so as to have similar α*-Bt*E IgE levels (all of them had α*-Bt*E IgE levels between 3.4 to 17.5 kIU/L, as determined by Pharmacia Immunocap System IgE FEIA (Pharmacia, Uppsala, Sweden). These 28 sera were divided into two groups, paired in accordance with α*-Bt*E IgE levels: 14 with positive *Bt*E SPT and 14 without any observable wheal formed when subjected to the *Bt*E SPT. Each serum from one group was paired with a serum from the other group that had similar α*-Bt*E IgE levels. Informed written consent was obtained from all individuals, and the study was approved by the Ethics Committee of the Maternity Hospital Climério de Oliveira, of the Federal University of Bahia, Brazil.

### *B. tropicalis *and *A. lumbricoides *extracts

The *B. tropicalis *was collected from bed dust in Salvador, Brazil, cloned and cultured with a fish food medium, at 25°C and 75% humidity. The mites were purified from the medium by flotation on a 5 M sodium chloride solution, followed by several washings by filtration in a 100 μm-pore polystyrene sieve with endotoxin-free distilled water. The washings were carried out until no food residues were seen under microscopy. The mites were lysed in 0.15 M phosphate-buffered saline, pH 7.4 (PBS), in an electric blender (Waring Commercial, Torrington, CN, USA). Lipids from the lysate were extracted by five or six ether extractions and discarded. The protein content of the *B. tropicalis *aqueous extract was determined by the Folin reagent method [28], and was subsequently stored at -70°C until use. The amount of *Bt*E used in the experiments was standardized by measuring its Blo t 5 content, using a commercially available capture ELISA kit (INDOOR Biotechnologies, Charlottesville, VI, USA). All used batches contained 30-40 ng of this allergen per μg of protein. An aqueous extract of *A. lumbricoides *was prepared from adult worms obtained from albendazole-treated infected children. The worms were washed with saline and crushed in an electric grinder (Bead-Beartas; Biospec, NC, USA), in the presence of PBS containing protease inhibitors (1 mM phenylmethanesulfonylfluoride, 2 mM ethylenediamine tetra-acetic acid, 50 μm tosyl phenylalanyl chloromethyl ketone and 50 μm tosyl-L-lysine chloromethyl ketone). The suspension was centrifuged at 4000 g for 15 minutes. The supernatant was stored at -70°C, and its protein content was determined by the Folin reagent method [28].

### Detection of serum anti-*Bt*E IgE antibodies

Microassay plate wells (Costar, Cambridge, ME, USA) were coated with *Bt*E by incubation with a *Bt*E solution containing 100 μg of protein.mL^-1^. After blocking the remaining free protein-binding sites with PBS containing 10% fetal bovine serum, serum samples, diluted 1:5 in PBS containing 0.05% Tween 20 and 5% fetal bovine serum, were applied to the wells in duplicates. Biotinylated anti-human IgE was added, followed by streptavidin-peroxidase (Pharmigem, San Diego, CA, USA). The reaction was developed using H_2_O_2 _and orthophenilenodiamine as substrate and chromogen, respectively (Sigma-Aldrich, St. Louis, ME, USA). The optical density to 480 nm light was measured in a plate reader spectrophotometer. Between all steps, the wells were washed three times with PBS containing 0.05% Tween 20 (PBS-T) followed by three times with PBS alone. The cut-off of the assay corresponded to the mean plus two standard deviations of the results obtained using sera from 10 individuals without history of allergy and with negative SPT reaction to the tested allergens.

### Detection of total serum IgE and calculation of anti-*Bt*E IgE/total IgE ratio

Total serum IgE levels were measured by capture ELISA. Briefly, microtiter plate wells (Costar 3590, flat bottom, polystyrene, high hinding; Cambrigde, MA, USA) were coated with 4 μg/mL of mouse anti-human IgE antibodies (Pharmigem, San Diego, CA, USA). The other assay steps were done as for the *α-Bt*E IgE assay described above. An IgE standard curve was obtained by using purified human IgE (Research Diagnostics Inc, Flanders, NJ, USA) and the results were expressed in international units (IU). A pool of sera from allergic patients who had positive SPT reactions to dust mite antigens was used as positive control. As negative control, an umbilical cord serum sample was used. An arbitrary α*-Bt*E IgE/total IgE ratio was calculated for each serum by dividing the value of α*-Bt*E IgE level that was detected in that serum (expressed in OD_480 nm_) by the value of total IgE level detected in the same serum (expressed as IU).

### Prevention of the binding of α*-Bt*E IgE to *Bt*E carbohydrate epitopes

In the indirect ELISA described above, some *Bt*E-coated wells were treated with 10 mM sodium metaperiodate (VETEC, Rio de Janeiro, Brazil) in 50 mM acetate buffer, pH 4.5, for 1 hour at room temperature in the dark, so that carbohydrates were oxidized to aldehyde. In control wells, the solid-phase *Bt*E was incubated only with acetate buffer. The wells were then washed once with 200 μL of sodium acetate buffer, and the formed aldehyde groups were reduced to alcohol through incubation with 100 μL of 50 mM sodium borohydride (Sigma-Aldrich, St Louis, MO) during 30 minutes at room temperature. After the incubation, the wells were washed three times with PBS-T and two times with PBS and used in the ELISA. The percentage of carbohydrate determinant-reactive α*-Bt*E IgE, in relation to the total α*-Bt*E IgE, was calculated in accordance with the formula: % of carbohydrate-reactive α*-Bt*E IgE = [1.0 - (mean OD of the results obtained in the assay of serum duplicates in the metaperiodate/borohydride-treated *Bt*E-coated wells/mean OD of the results obtained from serum duplicates assayed in control wells)] × 100, where OD = optical density.

### Blood cell cultivation and IL-10 detection

Venous blood samples were cultured at a dilution of 1:4 in RPMI (Gibco, Auckland, New Zealand) containing 10 mM glutamine (Sigma-Aldrich, St. Louis, MO, USA) and 100 μg/mL of gentamicin (Sigma-Aldrich, St. Louis, MO, USA). The cultures were performed within 6 hours of the blood collection, in a humidified atmosphere, with 5% CO_2_, at 37°C, for 24 hours. The IL-10 concentrations in cultures with non-stimulated blood cells and with blood cells that were stimulated by *Bt*E (40 μg/mL)*, Al*E (10 μg/mL), bacillus Calmette-Guérin (BCG) lysate (62.5 μg/mL; Fundação Ataulpho de Paiva, Rio de Janeiro, Brazil) or pokeweed mitogen (3.125 μg/mL; Sigma-Aldrich, St. Louis, MO, USA), were measured using commercially available anti - IL-10 antibodies and a standard curve constructed with recombinant IL-10, by sandwich ELISA, following manufacturer's instructions (BD Pharmingen San Diego, CA, USA). The detection range was 31.25 to 500.00 pg/mL.

### Investigation of cross-reactivity of *B. tropicalis *and *A. lumbricoides *antigens with serum IgE

The presence of IgE antibodies that cross-reacted with *B. tropicalis *and *A. lumbricoides *antigens was determined by a competitive ELISA. Briefly, different concentrations (0.3 to 300 μg/mL) of *Al*E, or the same volume of PBS, were incubated with the sera diluted at 1:2.5 overnight at 4°C. α*-Bt*E IgE were semi-quantified in these treated sera in a final dilution of 1:5, as described above. As a control for the specificity of the reduction in α-*Bt*E IgE levels, the concentration of total IgE was determined in the same sera. The proportions of α*-Bt*E IgE and total IgE levels that were reduced by incubation with *Al*E were calculated comparing the absorbance obtained in control sera (sera not pre-incubated with *Al*E) and in sera pre-incubated with *Al*E. Results were expressed in percentage of reduction, calculated as follows: percentage of reduction in α*-Bt*E IgE or total IgE level = [1 - (mean OD detected in the assay of duplicates of serum that had been pre-incubated with *Al*E/mean OD detected in the assay of untreated serum duplicates)] × 100.

### Statistical analysis

All statistical analyses were performed using the SPSS Version 13.0 program (SPSS Inc., Chicago, IL, USA). The Shapiro-Wilk test was used for verification of the data normality. The statistical significance of differences between the groups was assessed by the Mann-Whitney's U test. The Spearman's correlation between serum α*-Bt*E IgE level and the average diameter of SPT reactivity was calculated. A p value equal to or less than 0.05 was considered statistically significant.

## Abbreviation List

SPT: Skin prick test; *Bt*E: *B. tropicalis *extract; α*-Bt*E IgE: Anti-*B. tropicalis *extract IgE antibodies; *Al*E: *Ascaris lumbricoides *extract; IL-10: Interleukin 10

## Authors' contributions

**JCMP **conducted the experiments under the guidance of **NMAN **and drafted the manuscript. **SBJ **helped in the design and in the conduction of the experiments. **RVV **performed all statistical analysis and reviewed the manuscript. **MLB **coordinated the field studies. **LCPC **helped in the design of the experiments and carefully reviewed the manuscript. **NMAN **conceived and designed the study and coordinated the research team. All authors read and approved the manuscript.
